# Application of mesenchymal stem cell sheet for regeneration of craniomaxillofacial bone defects

**DOI:** 10.1186/s13287-023-03309-4

**Published:** 2023-04-07

**Authors:** Behnaz Banimohamad-Shotorbani, Sonia Fathi Karkan, Reza Rahbarghazi, Ahmad Mehdipour, Seyedhosein Jarolmasjed, Sepideh Saghati, Hajar Shafaei

**Affiliations:** 1 Department of Tissue Engineering, Faculty of Advanced Medical Sciences, Tabriz University of Medical Sciences, Tabriz, Iran; 2 Immunology Research Center, Tabriz University of Medical Sciences, Tabriz, Iran; 3 Department of Advanced Sciences and Technologies in Medicine, School of Medicine, North Khorasan University of Medical Sciences, Bojnurd, Iran; 4 Stem Cell Research Center, Tabriz University of Medical Sciences, Tabriz, Iran; 5 Department of Clinical Sciences, Faculty of Veterinary Medicine, University of Tabriz, Tabriz, Iran; 6 Department of Anatomical Sciences, Faculty of Medicine, Tabriz University of Medical Sciences, Tabriz, Iran

**Keywords:** Cell sheet technology, Craniomaxillofacial defects, Bone regeneration, Tissue engineering

## Abstract

Bone defects are among the most common damages in human medicine. Due to limitations and challenges in the area of bone healing, the research field has turned into a hot topic discipline with direct clinical outcomes. Among several available modalities, scaffold-free cell sheet technology has opened novel avenues to yield efficient osteogenesis. It is suggested that the intact matrix secreted from cells can provide a unique microenvironment for the acceleration of osteoangiogenesis. To the best of our knowledge, cell sheet technology (CST) has been investigated in terms of several skeletal defects with promising outcomes. Here, we highlighted some recent advances associated with the application of CST for the recovery of craniomaxillofacial (CMF) in various preclinical settings. The regenerative properties of both single-layer and multilayer CST were assessed regarding fabrication methods and applications. It has been indicated that different forms of cell sheets are available for CMF engineering like those used for other hard tissues. By tackling current challenges, CST is touted as an effective and alternative therapeutic option for CMF bone regeneration.

## Introduction

It has been accepted that tissue engineering (TE) is an interdisciplinary field to find appropriate biological substitutes for restoring injured tissues. For efficient reconstruction and mimicking functional and physical properties of tissues, TE uses three distinct elements as follows: heterogeneous stem cell population, suitable biomaterials, and varied growth factors [1, 2].

Among several stem cell types, mesenchymal stem cells (MSCs) are attractive cell sources for regenerative purposes because of their appropriate differentiation capacity and secretion of diverse soluble factors [3, 4]. MSCs are commonly isolated from different tissues such as adipose tissue, umbilical cord blood, bone marrow, and skeletal muscles. Adipose (AD-MSCs)- and bone marrow-derived MSCs (BM-MSCs) are two popular and most available cell sources for bone TE [5]. Although MSCs are a great stem cell source in the area of TE, a great plethora of studies has shown that MSCs isolated from different tissues possess variable degrees of regeneration capacity. For example, human ethmoid sinus mucosa-derived MSCs (hES-MSCs) display superior colonization properties and proliferation compared with maxillary sinus-derived MSCs [5]. In this regard, the selection of a suitable stem cell type can increase the possibility of osteogenesis and bone healing [6].

A comparison of different sources of MSCs [BM-MSCs, AD-MSCs, umbilical cord-derived MSCs (UC-MSCs)] indicated that AD-MSCs and BM-MSCs exhibit higher osteogenic potential in in vitro conditions related to UC-MSCs, while angiogenic capacity is more evident in AD-MSCs and UC-MSCs [7]. Data confirmed relatively similar osteogenic properties for these cells inside the body [7]. The existence of prominent osteogenic capacity in UC-MSCs and AM-MSCs is associated with a lower proliferation rate [8]. It should not be neglected that the occurrence of specific physiological and pathological conditions can affect the reparative properties of MSCs. For instance, the osteogenic capacity and proliferation of human and rodent BM-MSCs are reduced by aging [9, 10]. The application of MSCs in bone-related pathologies includes two main strategies. It is thought that MSCs can expedite the regeneration of injured bones via differentiation and secretion of several growth factors, and at the same time, properties like immunomodulatory, anti-inflammatory, and pro-angiogenesis activities can intensify the osteogenic capacity of MSCs [11].

CMF bone tissue supports numerous facial features such as pronunciation and mastication [12]. Compared to other appendicular bones, CMF basic discords include problems associated with endochondral and intramembranous ossification. Therefore, the same therapeutic strategies would not be suitable for both skull and long bones [13]. Usually, pathological injuries, even minor defects, related to maxillofacial bone are intricate in morphology compared with those that generally occurred in orthopedics [14].

Up to now, different strategies have been used for the regeneration and reconstruction of CMF bone injuries [15–17]. Cell-based therapies are at the center of attention for CMF bone regeneration [18]. Lendeckel et al. applied non-differentiated autologous AD-MSCs with fibrin glue to reconstruct calvaria with multi-fragment fractures in a seven-year-old girl. They also used polylactic acid (PLA)-based resorbable macro-porous mesh to fix the graft for three months [19].

Besides the efficacy of cells and different substrates in osteogenesis, introducing transplant cells in a specific structure can accelerate the healing procedure [20]. CST and other cellular sheets approaches are touted as scaffold-free methods containing natural extracellular matrix (ECM) but lack some problems related to the cell suspension seeding methods [21, 22]. Considering the importance of CMF regeneration and the high prevalence of CMF defects, we aimed to collect some experiments related to the application of CST in the regeneration of hard tissue, mainly in the CMF region.

## CMF defects: current methods and challenges

Bone tissue is considered the most extensively compact tissue that is continuously remodeled during life by osteoclasts and osteoblast activities [10, 23]. To date, CMF bone defects remain not only a considerable challenge for health but also for restoring functional esthetic façades and arise from several etiologies and congenital acquired deformities, such as degenerative diseases [15, 23], congenital malformations [5, 23], traumas [1, 5, 24], tumor resection [5, 23], bone atrophy following a tooth extraction [14], inflammation [1], and wrong surgical proceedings [23]. These features may lead to non-healing bone defects that entail fast and on-time maxillofacial bone reconstruction [1, 5, 14, 23–25]. It should be noted that spontaneous calvarial re-ossification would be successful only in babies younger than two [26]. Besides, the activity of mastication-related muscles provides a high amount of strain, and stress tolerated by CMF bones can increase the complexity of pathological conditions [12]. Due to the proximity of mastication-related muscles, maintaining the functional integrity of craniofacial bone and appropriate reconstruction at a high vascularization degree is a critical challenge in bone TE [12].

Small bone defects left without treatment that could not heal over their life are termed critical size defects (CSD) [27]. Restoration of extensive bone defects is essential in maxillofacial, oral, plastic, orthopedic, and reconstructive surgery. Most challenges and difficulties in the treatment of bone defects are associated with the application of suitable biomaterials designed using specific devices to promote the bone healing procedure and fill the missing tissue [1].

The clinical methods used to reconstruct CMF bone tissue could be divided into three main categories: bone grafting, biomaterials, and cell-based therapies [28]. Each modality has its advantages and limitations. Insufficient capacity for bone regeneration does necessitate bone grafting. Autologous bone grafts are one available option for surgeons and are touted as a gold standard modality for the restoration of CMF structure. Because of the existence of difficulties with bone grafting, researchers have focused on the development and application of other approaches, such as cell-based therapies [14, 23, 29]. Insufficient quantity of bone stock, variable effectiveness, immune reactions, accessibility, the possibility of infections, and morbidity are the main considerable limitations related to autologous and allogenic bone grafts [1, 28, 30, 31].

TE is an alternative approach for generating bone-like transplants to reduce or eliminate the demands for bone tissue grafting from a patient's secondary site [23, 32–34]. In autologous bone grafting, accessibility, mobility of donors on harvesting sites, unnecessary surgery, and anesthetic times are problematic issues [5, 29]. In the TE area, both allogeneic and xenogeneic materials have been commonly used for the acceleration of bone regeneration [2]. Limitations such as inaccessibility, infection probability, stableness, immunological reaction, storage, and unexpected quantity of graft resorption remained the most problematic issues in the application of biomaterials [15, 35].

During the past decades, various substrates such as ceramics, metal alloys and meshes (titanium mesh), demineralized matrix pastes, different polymers, porous hydroxyapatite (HA) materials, and cell-based approaches have been used as artificial grafts. Lack of osteoinductive features, problems related to the prosthetic material/bone interface, immunogenic clinical response, and removal of adjacent bone is prominent current limitations in the application of artificial materials [35, 36]. Recent approaches are based on the application of decellularized ECM to mimic the native bone ECM for efficient regeneration [37]. Along with various biomaterials, numerous phytocompounds like Danshen, Ge Gan, and Cissus quadrangularis extracts have been applied to improve hard tissue regeneration [38, 39]. Biomimetic scaffolds or implants are alternative options for CMF bone reconstruction [12]. However, incomplete biodegradation rate, prominent immunological response, lack of appropriate vascularization [12], foreign body reaction, chronic sinus mucosa swelling, fragmentation, infection [40], displacement of implant, epidural hematoma, and cerebrospinal fluid leak [41] have been reported in terms of biomimetic scaffold application.

## Scaffold-free CST

**Fig. 1:**
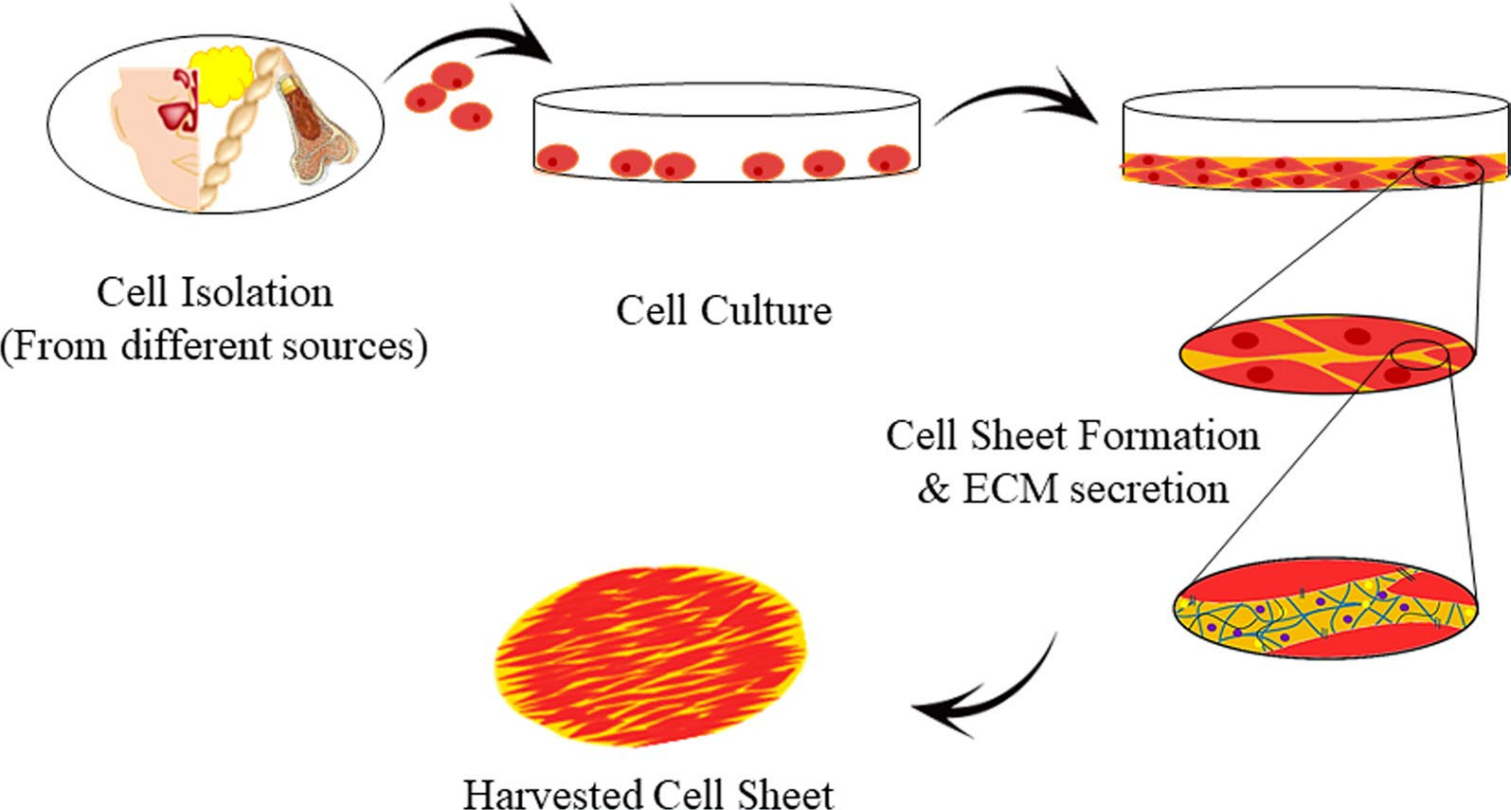
Schematic illustration of MSC sheet formation. The isolated MSCs from different sources are cultured for certain periods (please see Table 1). Cells generate single-layer or multilayer composite with intact ECM (sheet). MSC sheets can be harvested using several techniques such as electro-, thermo-, pH-, photo-responsive systems, mechanical force, and enzymatic treatment (Designed by office 2019)

Several research teams have promoted the regeneration of bony tissues using the cellular sheet [42, 43]. As an innovative method, CST could recreate a biological microenvironment similar, not completely but in part, to a target site [1, 5]. In this approach, cultured cells can be picked as integrated sheets without using normal proteolytic enzymes while cell-to-cell junctions are maintained (Fig. 1) [1]. Studies have proved the deposited ECM in the basal surface of the cell sheet compartment [1, 44].

Along with the disadvantages of biomimetic scaffolds inside the body, cell sheets may be a suitable regenerative approach with very low immune responses that can dictate in vivo-like phenotype for transplant cells with suitable angiogenesis [12]. Blood nourishment is a vital element for living tissues and bone remodeling procedures [45]. Compared to the cell suspension strategy, CST can notably lead to better cell retention, higher microvascular density, and juxtacrine interaction between the transplant and host cells within the interface area [37, 46]. Consequently, CST offers various considerable advantages and can circumvent several previous issues related to scaffolds. Cell sheets are routinely harvested by several methods, such as the application of thermo-responsive substrates at the bottom of cell culture dishes [44, 47], and the detachment of a single cell layer using mechanical forces (scraper) or light irradiation [14, 48]. Therefore, different responsive systems for cell sheet harvesting, such as electro-, thermo-, pH-, and photo-responsive systems, have been developed until now [49]. When cells are detached by enzymatic solutions such as trypsin or collagenase, considerable cell damage is possible, resulting in a low survival rate [50].

As mentioned above, mechanical retrieval and thermo-responsive plates beyond polymers are two primary methods for collecting cell sheets [10]. Data indicated intact underlying ECM, high-rate cell viability, and tissue-like behaviors in recovered cell sheets using these approaches. Besides, maintaining cell-to-cell and cell-to-ECM interaction can provide essential signaling pathways required for proper cell bioactivity. Immunohistochemical studies have confirmed the presence of ECM components such as fibronectin, laminin, and cell junction β-catenin in the sheet cell layer, which are essential for cell attachment and functionality [51]. It is believed that cells within the sheet structure can appropriately sense mechanical signals. Compared to a 3D culture system using scaffolds, CST benefits from several advantages [52]. The distributions of growth factors are acceptable, and most cells can be stimulated in a coordinated manner [52]. The activity of surface receptors such as glucose transporter-1, Na^+^/K^+^-ATPase, sodium–glucose-linked transporter-1, neutral endopeptidase, dipeptidyl endopeptidase IV, and aquaporin-1 are normal [53]. It is believed that the integrated ECM under the cell sheets could act as a natural tissue adhesive substrate and eliminate the necessity for suture, allowing sheets to attach rapidly to the tissue's target site without invasive manipulations [50–53]. All these events can occur using the intact ECM on the basal surface cell sheets [54]. Commensurate with these descriptions, the preservation of ECM in CS helps the transplant cells to regulate homeostasis, proliferation, and migration and provides crucial clues for mechanical support during the transplantation [5, 55, 56].

In the context of bone tissue structure, cell sheets can be used as periosteum [57]. Application of MSC sheets with supportive ECM circumvents disadvantages of cell-based therapies, such as lack of appropriate migration and leaking of transplant cells after injection into the injured sites [10, 52, 57].

The addition of distinct supplements, such as ascorbic acid, promotes the synthesis and secretion of ECM components and prevents the progression of age-related changes in cells within the sheet structure [44, 58, 59]. Akahane and colleagues declared that the exposure of cells to dexamethasone (Dex) and ascorbic acid affects the expression of specific genes associated with ECM construction and sheet formation [60]. Later studies on the role of Dex and ascorbic acid on BM-MSCs indicated that both factors allow the harvesting of cultured cells in a single cell layer by improving the mechanical integrity after induction of proteoglycan and type I collagen synthesis. Preparation and/or induction of integrated substrate synthesis can increase cell number, which seems a practical approach in complex tissue reconstruction like osteonecrosis, nonunion, and union bone defects [61]. By the stimulation of type I collagen, laminin, and fibronectin synthesis, ascorbic acid promotes the osteogenic differentiation of cultured cells [61, 62]. Likewise, the expression of stemness genes such as OCT-4, Sox-2, and TERT was upregulated in MSC sheets after exposure to ascorbic acid [44]. Langenbach and Handschel previously indicated that Dex could induce BM-MSC osteogenic differentiation by the expression of MKP-1 and osteocalcin (OCN) and dephosphorylation of Runx2 [61, 62]. It seems that incubation of cells in a culture medium supplemented with the combination of Dex and ascorbic acid yields efficient sheet formation culture systems compared to that from Dex or ascorbic acid alone [61]. In addition to certain components and stimulatory factors, the application of ECM components can trigger the formation of a cell sheet. For instance, gelatin enhances cell proliferation and upregulates the expression of the bone morphogenetic protein (BMP)-7 in the differentiated sheets [63]. It is touted that the existence of a specific tripeptide motif (arginine–glycine–aspartic acid) in the backbone of gelatin improves cell adhesion properties [63, 64].

According to previous studies, the roles of cell sheets in bone engineering containing exogenous constructs can be summarized below: (i) acting as cell carriers; (ii) restricting the progression of fibrous connective tissues in the osteogenic microenvironment; and (iii) developing periosteal and endochondral osteogenesis [50, 65, 66]. Up to now, several cells such as AD-MSCs, BM-MSCs, periodontal ligament-derived cells, dental follicle cells, and gingival margin-derived cells have been applied for sheet formation, and data confirmed their restorative properties in bone, corneal epithelium, periodontal, and myocardial tissue [5, 67]. Compared to single-cell sheet treatment, multilayer MSC sheets yielded better osteochondrogenic capacities. The culture of MSCs on stacked sheets composed of methyl cellulose and poly(N-isopropyl acrylamide) [MC and PNIPAAm] increased the expression of BMP-2, alkaline phosphatase (ALP), OCN, and VEGF at tendon–bone interface (TBI) [68]. For obtaining better regenerative outcomes, Berntsen et al. used stacked tenogenic and osteogenic hAD-MSC sheets. Juxtaposed tenogenic and osteogenic hAD-MSC sheets with an integrated multilayer construct increase simultaneously several biomarkers associated with tendon, mineralized fibrocartilage, and bone tissue. Data indicated a spatial gradient of RUNX2 expression. In the osteogenic sheet, the expression of OCN and osterix were increased coinciding with the transcription of tenomodulin and scleraxis at the tenogenic cell sheet. Of note, endochondral ossification was evident in the engineered interface which is indicated by Indian Hedgehog and Type X collagen [69]. Simultaneous application of BM-MSC sheet with an acellular structure is another strategic approach to achieve triple biomimetic TBI.

However, varied cell types can affect the behavior of the final cell sheet structure. Recently, Nakao et al. have investigated differences between MSC sheets produced using various cell sources like AD-MSCs, BM-MSCs, and human umbilical cord MSCs (hUC-MSCs) [70]. Based on data, adhesion, and migration of MSCs were similar. However, some differences were found regarding cytokine secretion and proliferation capacity. On the surface of thermo-responsive cell culture dishes, BM-MSCs exhibited lower adhesion compared to AD-MSCs and UC-MSCs. Forty-eight hours after seeding, the number of AD-MSCs and UC-MSCs was higher than BM-MSCs. However, BM-MSCs showed the most potent generation rate. As a correlate, the selection of a suitable MSC source with prominent cytokine secretion capacity is integral to the successful transplantation process [70].

Akahane et al. investigated the relationship between donor age and the osteogenic potential of osteogenic matrix cell sheets (OMCS). They found a similar level of osteogenesis capacity and bone formation in both old and young donor cells, indicating the fact that OMCS can be considered an efficient method in bone healing even in aged patients [10]. Emerging data have indicated that cell sheets can improve the paracrine activity of MSCs after placing at the site of injury [71]. It was indicated that the secretion of exosomes via mouse BM-MSCs sheet promoted neural stem cell differentiation, axonal regeneration, and synaptogenesis in a mouse model of spinal cord injury [71]. These data show that cell sheets can improve the regenerative properties of MSCs via the induction of differentiation and paracrine activity.

## Cell sheet in CMF defects

**Fig. 2:**
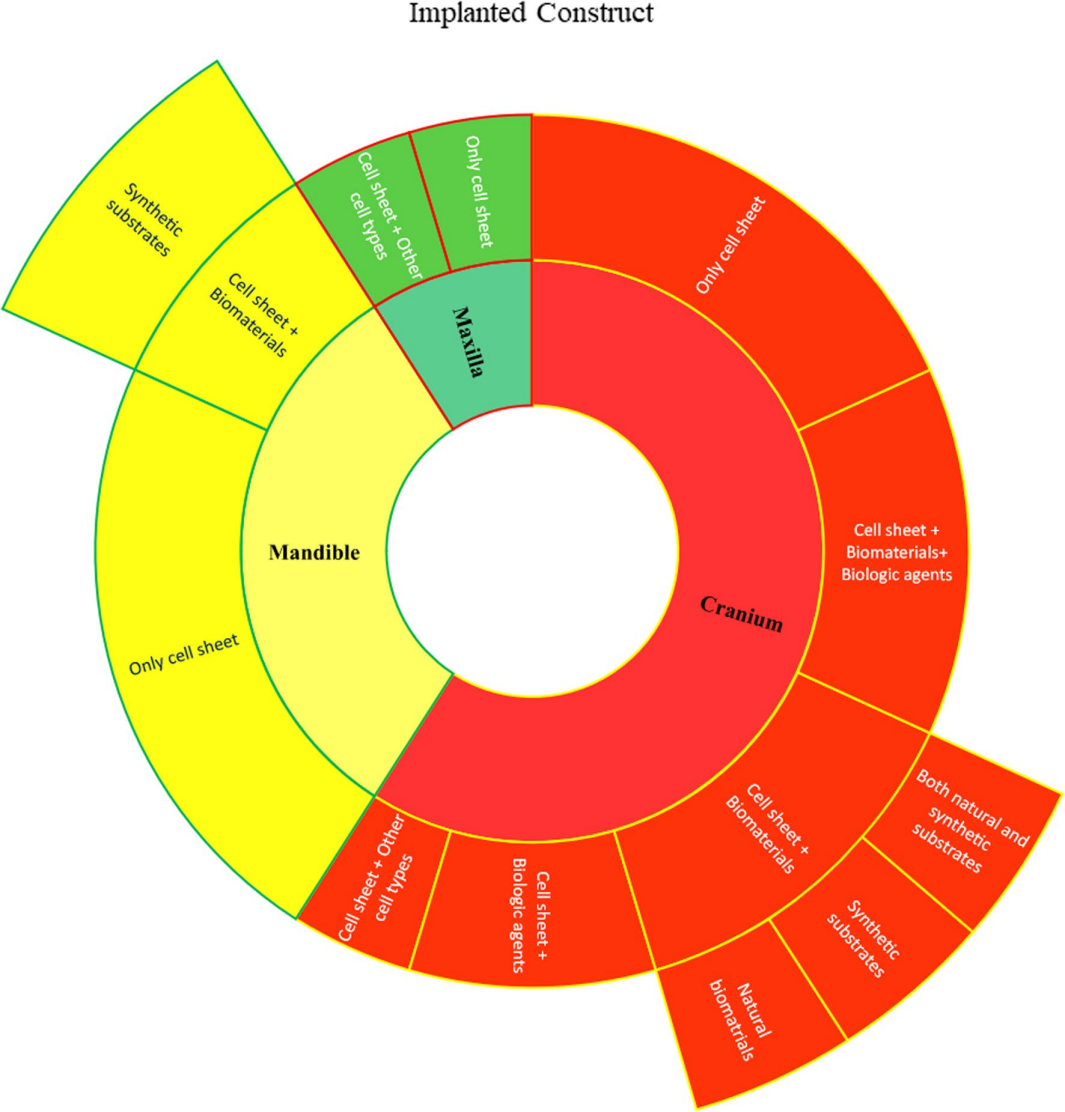
Application of MSC sheet alone in combination with other substrates and growth factors for the acceleration of osteogenesis in the mandible, maxilla, and cranium (Designed by office 2019)

Recently, allogeneic monolayer and heterogeneous multilayer cell sheets have been developed for TE purposes. Cell sheets have been used to construct tissues such as bone, mucosa, cornea, and myocardium [50, 72–74]. In some studies, CST was combined with other biomaterials for bony tissue reconstruction, such as spinal cord interbody fusion [75]. Figure 2 and Table 1 show sheet-based constructs for CMF bone regeneration.

**Table 1:** Summary of studies related to the application of MSC sheets for the regeneration of the calvarial, mandible, and maxillary bone defects

Region of defect	Defect size	Animal	Construct	In vitro culturing time of sheet formation	Ascorbic acid	Dex	Harvesting or transferring method	Post-implantation time	In vivo tests for cell sheet evaluation	References
Cranium	8 mm diameter (CSD)	8-week-old female Sprague Dawley rats	Human ES-MSC sheets	7 days with Vitamin C and osteogenic inductive medium	100 mg/mL	–	A sterile forceps (physical harvesting)	8 weeks	– Micro-CT – Sequential fluorescent Labeling – Histological and histomorphometric observation – Immunohistochemistry	[5]
Cranium	15 mm diameter (CSD)	3- to 5-month-old New Zealand rabbits	BM-MSC sheets + nano-HA + PRF	14 days	50 µg/ml	10 nM	A cell scraper (physical harvesting)	8 weeks	– Histological and histomorphometric analysis – 3D Computerized tomography	[1]
Cranium	15 mm diameter (CSD)	3-month-old New Zealand rabbits	BM-MSC sheets	14 days	50 µg /ml	10 nM	A cell scraper (physical harvesting)	8 weeks	– Micro-CT analysis – Histological analysis of bone – Van Gieson staining	[76]
Cranium	15 mm diameter (CSD)	Adult New Zealand rabbits	BM-MSC sheets + demineralized bone matrix	14 days	50 µg/mL	100 nM	A cell scraper (physical harvesting)	6 and 12 weeks	– Radiographic analysis – H & E staining	[27]
Cranium	5 mm diameter (CSD)	6-week-old male Sprague Dawley rats	Multilayer BM-MSC cell sheets + PLLA/gelatin electrospun mesh (CSMs)	Cells were cultured for 7 days to generate sheets and then four monolayer CSMs were stacked layer-by-layer and incubated for another 3 days to construct multilayer CSMs	0.05 mM	1 × 10^−8^ mM	The cell sheet was not detached because it was generated on the mesh surface	4 and 12 weeks	– Histological analysis (H&E and Masson’s trichrome staining) – Immunohistochemical staining (OCN and OPN) – Micro-CT	[77]
Cranium	6.8 mm diameter	10-week-old male F344 rats	Periosteal cell sheets	5 days and cell sheets were detached using 0.25% Trypsin–EDTA solution and then cells were cultured for additional 10 days	–	–	Reduction of temperature below 20 °C for 30 min (physical harvesting)	24 weeks	– Micro-CT – Histology	[78]
Cranium	5 mm diameter	Adult female New Zealand rabbits	Alveolar BM-MSC sheets	10 days	20 µg/mL	-	–	4 and 8 weeks	– Micro-CT – Histology analysis – Immunofluorescence analysis	[79]
Cranium	5 mm diameter	8-week-old male Sprague Dawley rats	AD-MSC sheets + Bone Granule Complex (with/without) Semaphorin 3A injection	7 days	-	-	-	4 or 8 weeks	– Micro-CT – Histomorphological analyses	[80]
Cranium	5 mm in diameter	12- to 13-week-old male Sprague Dawley rats	BM-MSC sheets + PLGA/HA Scaffolds + human BMP-2 vectors	2 weeks	-	-	A cell scraper (physical harvesting)	4 and 8 weeks	– Micro-CT – Histological examination – qRT-PCR analysis of hBMP-2 expression	[81]
Cranium	1.6 mm diameter	6- to 8-week-old male C57BL/6j or NOD/SCID mice	Human BM-MSC sheets + IFN-γ	4 days	50 µg/mL	-	Scratched using a micropipette tip (physical harvesting)	7 and 28 days	– Micro-CT – Histological analysis	[56]
Cranium	15 mm diameter (CSD)	3-month-old New Zealand rabbits	BM-MSC sheets + PRF	2 weeks	50 mg/mL	-	A cell scraper (physical harvesting)	8 weeks	– Micro-CT – Histological analysis	[82]
Cranium	10 mm diameter	3-month-old New Zealand rabbits	AD-MSC sheets + endothelial progenitor cells	2 weeks	50 mg/mL	100 nM	ND	8 weeks	– Micro-CT – Histological analysis	[24]
Cranium	8 mm diameter	Female Wistar rats	Differentiated rBM-MSC sheets + Porous β-TCP scaffolds	14 days + 7 other days incubation with induced endothelial-like cells	50 mg/mL	–	–	2, 4, and 8 weeks	– X-ray – Micro-CT – Histological analysis – Immunohistochemistry staining	[59]
			Osteogenic cell sheet + Porous β-TCP scaffolds	21 days	50 mg/mL	10 nM				
Mandible	5 mm diameter	Beagle dogs	AD-MSC sheets	1 week	82 µg/mL	–	By reducing the room temperature to (physical harvesting)	6 weeks	– Micro-CT – Photographic image analysis – Immunohistochemical analysis – Histological analysis	[11]
Mandible	Vertical osteotomies (the authors did not give exact size)	6- and 8-month-old Male New Zealand rabbits	BM-MSC sheet fragments	2 weeks	50 g/ml	100 nM	A cell scraper (physical harvesting)	3 and 6 weeks after injection	– Micro-CT – Radiographic DXA examinations – Histological and Histomorphometric analyses	[52]
Mandible	Large defect (the authors did not give exact size)	14-month-old male mongrel dogs	PLGA scaffolds with or without BM-MSC sheets	7–10 days	–	–	By reducing the temperature below 20˚C for 60 min (physical harvesting)	4, 8, 12, and 16 weeks after surgery	– X-ray analyses – H & E staining – Bone hardness analysis	[53]
	A trapezoid defect (the authors did not give the size)	14- to 23-month-old mongrel dogs	PLGA with and without canine stem cell sheets after osteoblast induction	12 days	–	–	The culture dish was placed in the calorstat for 25 min	3, 9, and 12 weeks	– X-ray imaging – Histological observation	[83]
										Mandible
Mandible	Not given	4-month-old female, domestic pigs	Triple-layer BM-MSC sheets	Two consecutive days of seeding, 2 other days of incubation	–	–	By reducing the temperature below 20˚C for 1 h	6 weeks	– Micro-CT – Histological analysis – Immunohistochemical analysis	[84]
Mandible	Not given	15-week-old syngeneic rats	folded BM-MSC sheets with an average diameter of 2 mm	Until reaching confluence	0.28 mM	10 nM	A cell scraper (physical harvesting)	2, 4, and 8 weeks	– Micro-CT – Histological (H & E) analyses – Toluidine blue staining	[14]
Mandible	Not given	3-month-old female, domestic pigs	Triple-layer BM-MSC sheets	48 h	–	–	Reducing the room temperature for 1 h	6 weeks	– CT – Mineral apposition analysis – Histomorphometric analysis	[85]
Maxillary	Not given	4-week-old females Sprague Dawley rats	Fluorescent-labeled BM-MSC sheets	7 days	82 µg/mL	–	Reducing the room temperature	2 weeks	– Micro-CT – Histological analysis – Immunohistochemical analysis	[86]
Maxillary sinuses	Not given	Female New Zealand rabbits	Differentiated and undifferentiated BM-MSC sheets, with nasal mucosa-derived cell sheets	2 days	–	–	Reducing the temperature below 20 °C for 30 min	4 weeks	– H & E staining – Immunohistochemical staining – SEM – TEM	[87]

CSD: Critical size bone defects; transmission electron microscopy: TEM; scanning electron microscope: SEM; hematoxylin and eosin staining: H & E staining; poly(lactic-co-glycolic) acid: PLGA; bone density scan: DXA; bone marrow mesenchymal stem cells: BM-MSCs; adipose-derived MSCs: AD-MSCs; platelet-rich fibrin: PRF; poly(L-lactide): PLLA; β-tricalcium phosphate: β-TCP; hydroxyapatite: HA

## Efficacy of cell source and passage numbers

**Fig. 3:**
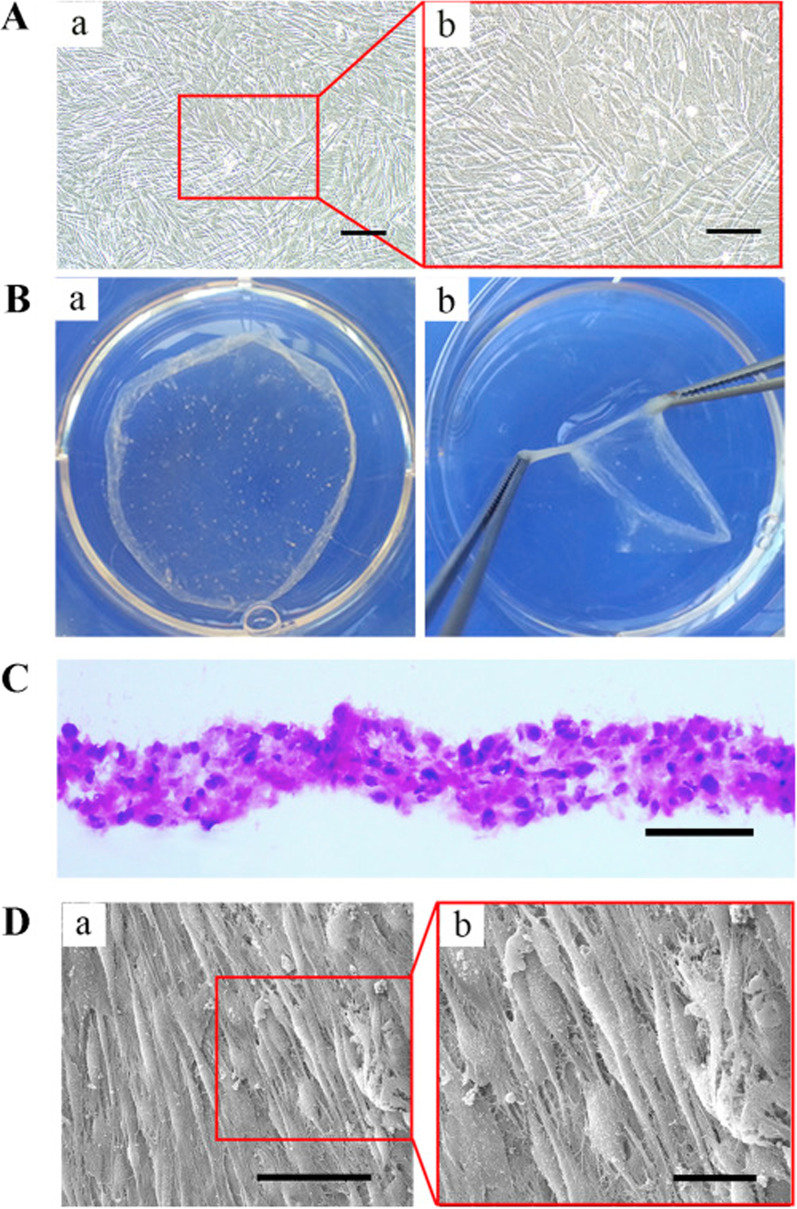
Bright-field assessment of hESMSC sheets with different magnifications (**A**: a = 500 µm and b = 200 µm). The cell sheets were detached from the temperature-responsive dish at a temperature below 20 °C (**B** panel a) and lifted using forceps (**B** panel b). H & E staining revealed the existence of 4–5 cell layers with an average thickness of 100–150 µm (**C**; scale bar = 200 µm). The ultrastructural analysis confirmed that compact cells are embedded in dense ECM (**D**: panel a = 50 µm; panel b = 20 µm). Copyright with permission [5], 2015, Biomaterials

To answer whether the source of cells would be an effective regenerative outcome, Liu et al. compared the osteogenic potential of alveolar and long BM-MSC sheets in rabbits with calvarial bone defects [79]. According to the data, sheets containing alveolar BM-MSCs yielded more osteogenic outcomes with prominent mineralization and higher newly formed bone-to-total-volume ratio (BV/TV) and significant expression of osteonectin, OCN, Runx2, osteopontin (OPN), and BSP genes compared to the group received long BM-MSCs [79]. The authors suggested that the application of alveolar BM-MSC sheets would be a hopeful approach to the regeneration of CMF bone injuries in clinical settings [79]. In an experiment conducted by Xie et al., human ethmoid sinus mucosa (ES)-MSCs and rat BM-MSCs sheets in the presence of polysebacoyl diglyceride were fabricated and examined in a rat model of calvarial defects [5]. To this end, different composites [ES-MSCs‒sheet‒BM-MSCs‒polysebacoyl diglyceride, BM-MSCs–sheet–BM-MSCs‒polysebacoyl diglyceride, BM-MSCs‒polysebacoyl diglyceride, polysebacoyl diglyceride] were fabricated and transplanted into the target injury site. Based on the data, it confirmed that hES-MSCs‒sheets aligned with BM-MSC‒sebacoyl diglyceride showed maximum osteogenic properties after transplantation into the injury sites. Also, this group had higher paracrine activity to release factors such as BMP-2, BMP-4, and bFGF in in vitro conditions (Fig. 3) [5]. It should not be forgotten that the number of cell passages can affect the quality and restorative capacity of the cell sheets. Kim et al. fabricated cell sheets from hUC-MSCs at different passages (from P4 to P12) on thermo-responsive culture dishes [51]. Based on the data, the proliferation properties of MSCs were reduced upon reaching passage 9 with inadequate cell-to-cell juxtacrine interaction. It confirmed that MSCs tended to form micro-sized cell aggregates with heterogeneous morphologies by the increasing number of passages in which passage 12 MSCs lost their properties to form a confluent single cell layer.

## Implantation of the cell sheet

### Fragmented cell sheet

Sheet pieces are eligible to initiate bone generation at different time points, with a higher peak load compared to integrated cell sheet transplantation. Related outcomes represented a considerable increment in bony junction using fragments of the BM-MSC sheet [52].

### Multilayer cell sheet

It has been shown that triple-layer BM-MSC sheets could appropriately stimulate local surface bone growth [84]. Data obtained indicated that autologous pig mandibular BM-MSC sheets could increase bone growth at the mandibular symphyseal surface [84].

## Cell sheet and biomaterials

The combination of CST with certain biomaterials can increase the regenerative potential of transplant cells [1, 5]. The presence of nanofibers meshes composed of poly-L-lactide (PLLA) and gelatin (PLLA/gelatin), not only improved and induced osteogenic differentiation of BM-MSCs but also remarkably enhanced new calcified bone formation [77]. Cell sheets in combination with PRF (platelet‑rich fibrin granules) and nano‑HA [1], poly(lactide-co-glycolide)/HA (PLGA/HA) scaffold [81], PLGA [53], beta-tricalcium phosphate (β-TCP) [59], and β-TCP/collagen-I [88] could be more effective in the regeneration of calvarial bone defects. It is postulated that the combination of a cell sheet with a 3D scaffold structure can yield more regenerative outcomes [53]. To this end, Du and co-workers cultured human BM-MSCs within the biodegradable β-TCP scaffolds and wrapped them simultaneously with BM-MSC sheets. This strategy reduces unwanted adipogenic and chondrogenic differentiation of cultured cells and increases osteogenic potential [89].

## Cell sheet and biological agents

It has been indicated that interferon (IFN)-γ stimulates immuno-suppressive properties of MSCs by the regulation of the indoleamine 2, 3-dioxygenase (IDO) enzyme [56, 90]. Studying the effect of IFN-γ on hBM-MSC sheets showed that the upregulation of IDO leads to HLA-DR expression and T cell inhibition in grafted regions, resulting in the induction of mice calvarial bone regeneration. It is thought that IDO can diminish the host immune response, thus inducing bone tissue regeneration in the mouse model [56]. The combination of the BM-MSC sheet and PRF increased the healing of the critical size calvarial bone injury in rabbits [82]. Also, local injection of the Semaphorin 3A (Sema3A) with AD-MSC sheet and Bio-Oss® bone granules increased the osteogenic outcomes. This strategy seems to be an exciting approach for osseous healing in rats with type 2 diabetes mellitus [80]. Kaibuchi and co-workers proved the efficacy of AD-MSC sheets in the healing of mandibular wounds in beagle dogs caused by Dex and zoledronate. Following the operation, many bacterial colonies and multi-nuclear Cathepsin K-positive cells were detached from the surfaces of the jaw bone on the control side, while a complete healing process in the mucosal wounds was achieved in all dogs that received MSC sheets [11].

## MSC sheet and angiogenesis

Among several important factors involved in bone healing, angiogenesis, and vascularization have critical roles [15, 91]. Efficient bone TE and regeneration require rapid neovascularization into the grafts [76]. Without tissue vascularization, the implants will not develop bone-forming units or lose their function over time [92]. Supplementation of pro-angiogenic factors, co-culture approaches, microsurgical techniques, and optimizing the scaffold structure are some ways to stimulate angiogenesis [93–97]. MSC secretome encompasses several angiogenic factors such as interleukin (IL)-6, FGF-2, VEGF, monocyte chemoattractant protein-1, and angiopoietin-1 [1]. It has been shown that hypoxic AD-MSCs could induce the growth of blood vessels by concurrent secretion of anti-apoptotic and angiogenic factors, like VEGF [98–101]. Thus, AD-MSCs have the potential to affect both osteogenic and angiogenesis (osteoangigoenesis) in sheet form [24, 102, 103]. The angiocrine and anti-apoptotic factors released by BM-MSC could regulate endogenous cell migration [81]. In BMP-2-mediated bone tissue regeneration, the paracrine activity of osteoprogenitors is considered a great modulator of neovascularization as well [99, 100, 104, 105].

**Fig. 4:**
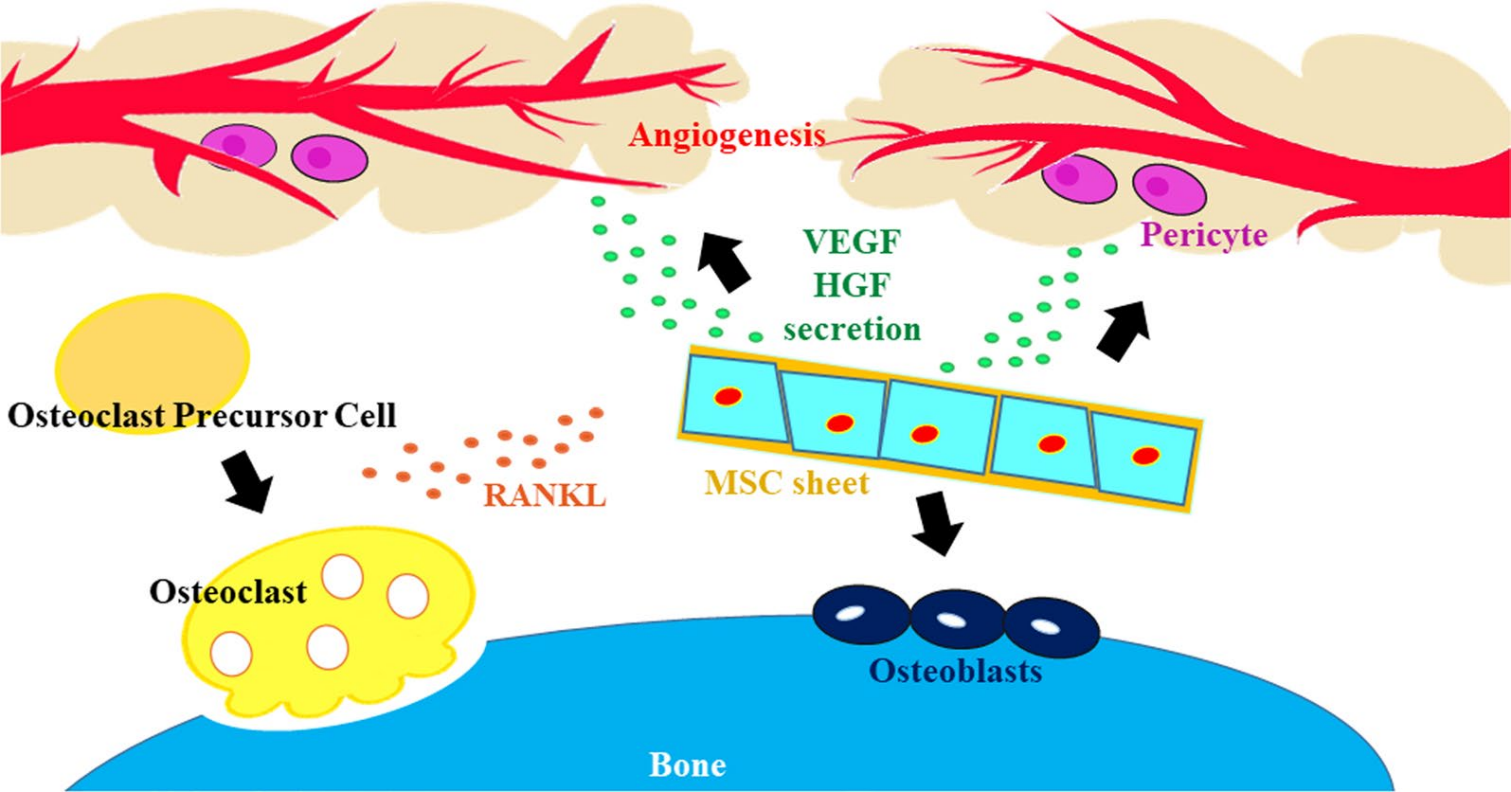
Possible restorative properties of MSC sheets in rats with BRONJ disease. Like MSC differentiation properties, the paracrine activity of MSCs within the sheet structure is an important factor to accelerate osteogenesis. It is believed that simultaneous activation of angiogenesis and osteogenesis by MSC sheet is critical to yield better regenerative outcomes. The release of VEGF and HGF promotes angiogenesis while RANKL secretion can orient the osteoclast precursor cells into osteoclasts and improves bone remodeling in rats with BRONJ disease (Designed by Photoshop version CS6)

**Fig. 5:**
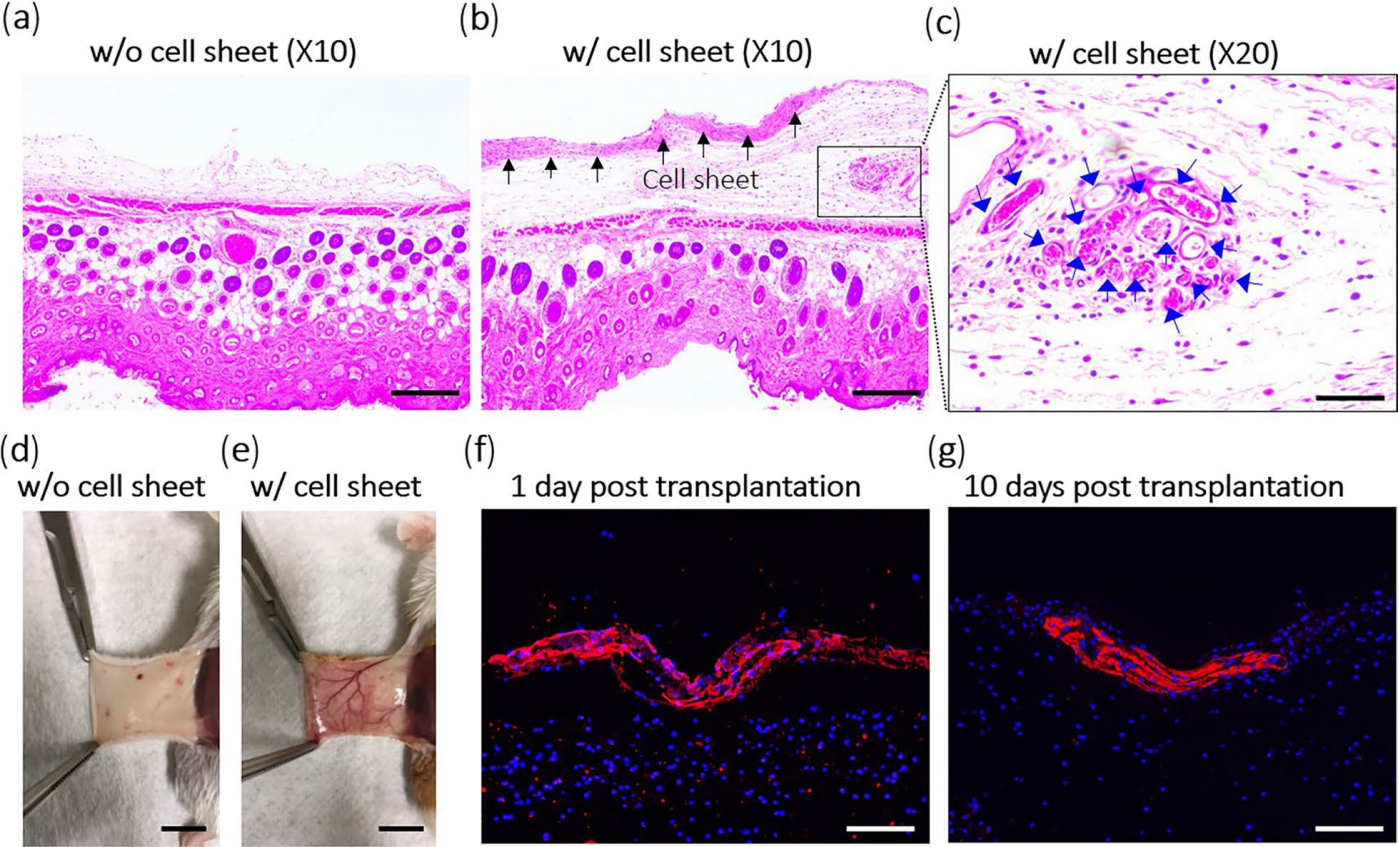
Xenogeneic transplantation of human umbilical cord MSC (hUC-MSC) sheets to immuno-deficient mice (**a**–**g**). H & E staining indicates the formation of numerous vascular units in mice that received xenogeneic hUC-MSC sheets (**b** and **c**) compared to the control group (**a**). The transplantation area is indicated with local vascularization (**d**). Immunofluorescence images confirmed that hUC-MSC sheets produce HGF on days 1 and 10 after being transplanted into the target sites within the cutaneous tissue (**f** and** g**). Black arrows = transplanted hUC-MSC sheet; blue arrows = blood vessels. Scale bars in panels **a** and **b** = 100 µm; scale bars in panels **c**, **f** and **g** = 50 µm; scale bars in panels **d** and **e** = 0.5 cm. Copyright with permission [51] 2019, Scientific reports

The angiogenesis potential of MSC sheets has been highlighted in different tissues, such as the skin and heart [42]. It was suggested that MSC sheets produce significantly higher VEGF levels compared to MSC suspension after being placed at the site of injury or ischemia [57, 68, 106, 107]. The release of specific factors such as HGF and VEGF stabilizes newly formed blood vessels following the differentiation of MSCs. Of note, a fraction of MSCs within the sheets acquires a pericyte-like phenotype (Fig. 4) [86]. Therefore, one can hypothesize that the release of angiocrine by MSCs is an important factor in the acceleration healing process in the early days [51]. Based on the data, the simultaneous application of materials with engineered cell sheets (MSC sheet implants) can simultaneously increase vascularization and osteogenic abilities [108]. In this regard, Nakano and colleagues differentiated developed rat BM-MSC sheets in the presence of osteogenic medium and placed the cells within the cylindrical shaped β-TCP scaffolds [109]. It was indicated that both vascular units and bone-forming units were seen at the center of β-TCP construct two weeks after transplantation into the rats, leading to improved new bone generation [109]. Molecular investigations revealed the expression of ALP, BMP-2, OCN, and VEGF-A. It is thought that the concurrent release of VEGF with osteogenesis factors like OCN and ALP around the fracture sites after transplantation of cell sheets can help with new bone formation and remodeling [110]. Similarly, Kim et al. confirmed angiogenesis, capillary formation, and newly generated blood vessels between host tissue and hUC-MSC sheets 10 days after implantation. They showed continuous and appropriate secretion of human HGF for ten days while in the absence of hUC-MSC sheets (MSC suspension), only a few tiny blood vessels are generated (Fig. 5) [51]. Likewise, nascent vascular units have been approved around MSC sheets in different animal models. In a study, allogenic GFP-tagged rat BM-MSC sheets were transplanted into rats with osteonecrosis of the jaw [86]. Immunofluorescence staining revealed that these cells can juxtapose to the periphery of vessels and exhibit pericyte marker CD146, indicating the active participation of transplanted MSCs in the arteriogenesis process [86]. To be specific, the differentiation of MSCs toward pericytes can stabilize the structure of newly generated vascular units [111]. Multiple cell transplantation is an interesting strategy to increase the angiogenic potential of stem cells. Using an MSC, endothelial cell, and CD146 pericyte co-culture system on PIPAAm thermo-responsive surfaces, Mendes et al. improved the osteoangiogenic capacity of fabricated MSC sheets [112]. They showed that endothelial cells and CD146 pericytes migrated successfully to the MSC layer and several vascular anastomoses with the production of type I collagen and osteocalcin [112]. In some studies, cell sheets have been used along with biodegradable scaffolds to stimulate vascularization. Kang and co-workers used β-TCP-covered biomimetic periosteum cell sheets for bone tissue regeneration [113]. The biomimetic periosteum was effective in osteogenesis and vascularization due to an appropriate spatial configuration. By incorporating a vascular bed or collagen micro-channels into stacked sheets of cells, the medium could be filled into the sheets to build new capillaries inside the perfusion bioreactor [92, 114, 115]. It should be noted that it is necessary to use appropriate doses of components to avoid the development of excessive blood vessel formation that could lead to pathological diseases such as vascular malformations [116], atherosclerosis, and proliferative retinopathy [24, 116].

## Immune response and cell sheet

Undoubtedly, the infiltration of various immune cells like Th1, Th2, Th17, and macrophages into the grafts is essential for tissue regeneration or wound healing [79]. Up to now, the immune-suppressive impact of MSCs is well proved in several reports. MSCs can secrete specific bioactive molecules that could avert unpleasant immune reactions and support the healing of damaged tissues [1]. Modulatory potential and anti-inflammatory effects of MSCs occur mainly via paracrine mechanisms [5]. It is shown that MSCs can exhibit anti-inflammatory effects in many diseases via the regulation of macrophage activity [87, 117]. As reported in several animal and clinical studies, spontaneous tissue healing might lead to difficulties, such as fibrosis and inflammation. Minimizing these responses could be possible using graft materials.

In most circumstances, treatment using allogeneic MSCs is considered due to the local immuno-suppressive activities of MSCs, where using autologous cells is restricted or even impossible [1]. A limitation of allogeneic cell therapy involves immunological properties and variable efficiency in patients due to cell transfer strategies. Therefore, researchers have tried to develop novel allogeneic cell sheets using hUC-MSCs with low antigenicity. Compared to several types of MSCs, such as BM-MSC and AD-MSC sheets, the UC-MSC sheets barely express MHC II antigens related to all immune rejection (Table 2). hUC-MSC sheets could be grafted immediately within 10 min of placement into the subcutaneous tissue in immune-deficient mice. The secretion of TNF-α from hUC-MSC sheets at passage 4 was notably lower than those of sheets at passages 6, 8, 10, and 12. Together, the passage number of cells is an important factor in immunomodulatory capacities [51, 70]. It has been indicated that cytokines such as IL-6, IL-10, TGF-β1, and PGE2 are associated with the anti-inflammatory properties of MSCs [118]. TGF-β1 can down-regulate the inflammatory reactions by damping macrophage movement and release of pro-inflammatory cytokines [119]. TGF-β1 exerts pleiotropic influences on processes including cell proliferation, migration, differentiation, and death [120]. Also, this factor can eliminate T-cell activity in response to exogenous antigens [121], suggesting that the levels of TGF-β1 may influence the survival of grafted MSCs. It was found that the production of PGE2 was moderately high in UC-MSCs and BM-MSCs as compared to AD-MSCs [86]. PGE2 is involved in the anti-inflammatory response by binding to its receptors, EP1eEP4 [70, 122]. Corradetti and co-workers indicated the infiltration of neutrophils into the grafts at the beginning of 1–4 weeks accompanied by monocyte recruitment [123]. In the latter steps, this pattern was turned into anti-inflammatory cells [124].

**Table 2:** Comparing the sources of MSCs. Cytokine expression from cell sheets with different cell sources including UC-MSCs, BM-MSCs, and AD-MSCs [70]

Cytokine (pg/ml per cell sheet)	Qualitative amount
HGF Level	AD-MSCs ˃ BM-MSCs ˃ UC-MSCs
TGF-β1 Level	BM-MSCs ˃ UC-MSCs ˃ AD-MSCs
PGE_2_ Level	BM-MSCs ˃ UC-MSCs ˃ AD-MSCs
IL-6 Level	UC-MSCs ˃ BM-MSCs ˃ AD-MSCs
IL-10 Secretion	AD-MSCs ˃ BM-MSCs ˃ UC-MSCs

Liu et al. reported that the immunoregulatory of alveolar BM-MSC and iliac BM-MSC sheets are similar with nonsignificant changes in the activity of Th1, M1, and M2 cells [79, 125]. Unlike this study, some authorities found that different MSC sources (BM-MSCs, AD-MSCs, and fibroblasts) within the cell sheets can exert varied anti-inflammatory properties [117]. It was indicated that AD-MSC and BM-MSC sheets stimulated the anti-inflammatory macrophage phenotype (M2) better than fibroblast sheets. Based on data, supernatants collected from AD-MSC sheets yielded higher CD206 surface markers, IL1 receptor antagonist (IL1RA), and chemokine (C–C motif) ligand 18 (CCL18) [117]. It has been shown that CD206, a mannose receptor, is upregulated in response to certain interleukins such as IL-4 and IL-13. CD206 may modulate signals induced by other receptors, such as Fc or Toll-like receptors [126]. Likewise, IL-1Ra acts as the IL-1 inhibitor by binding to the IL-1 receptor to protect tissues from inflammation-induced injuries [127].

## Other possible mechanisms involved in the regenerative potential of CS

As discussed above, MSCs can commit to several cell types and promote the regeneration of tissues via different mechanisms. In addition to osteoangiogenic and immune-modulatory properties, these cells exhibit osteoinductive, neurogenic capacities via engaging paracrine mechanisms [5]. After transplantation, cell sheets not only maintain cellular function and morphology but also can help the transplanted cells to adhere to the host tissues and secret growth factors [51].

**Fig. 6:**
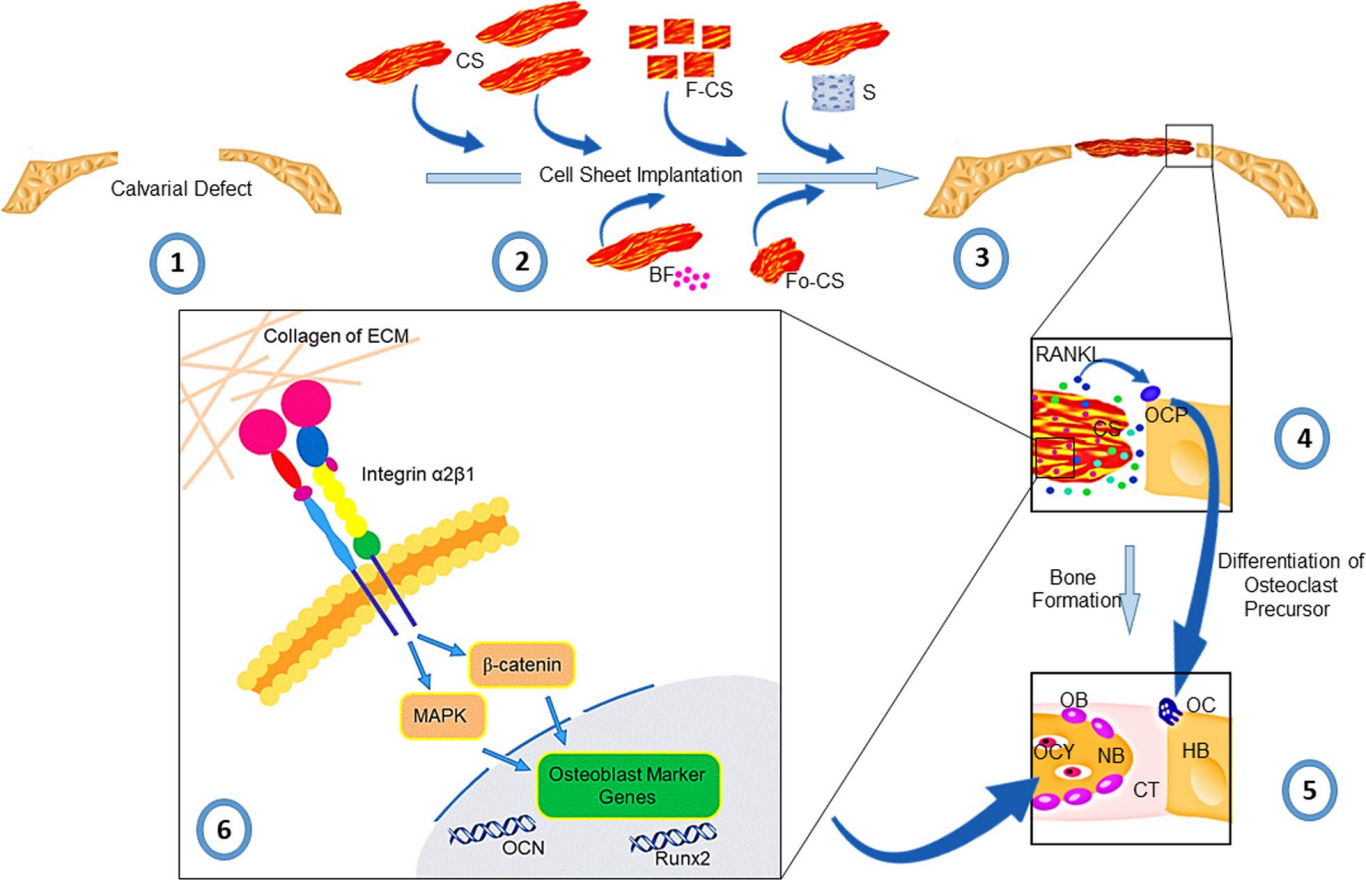
Cell sheet implantation approaches and subsequent molecular responses. CS could be used as mono- or multi-cell layered form, F-CS, and Fo-CS in the site of bone injury. It is suggested that CS can be applied in combination with S, and BF. After CS transplantation, implanted cells release several factors such as RANKL with the potential to increase osteoclast precursor cells differentiation into osteoclasts. Reciprocal interaction of ECM integrin and collagen with cells leads to the activation of relevant downstream signaling pathways such as MAPK and β-catenin, resulting in OB differentiation and increased bone formation. Cell sheets: CS; fragmented form: F-CS; scaffolds: S; biological factors: BF; folded form: Fo-CS; osteoclasts: OC; osteoblasts: OB; osteocytes in lacunae: OCY; connective tissue: CT; new bone: NB; and host bone: HB (Designed by Office 2019 and Photoshop CS6)

Kaibuchi et al. showed that the average number of osteoclasts was significantly higher in MSC sheets than in the control and suspended MSCs groups. Also, the secretion of receptor activator of nuclear factor ĸ-B ligand (RANKL) from MSCs could lead to the differentiation of osteoclast precursor cells to osteoclasts [86]. Xiao et al. suggested a plan to describe how ECM can regulate osteoblast differentiation [128]. They proposed that osteoblasts phenotype is acquired after the promotion of close contact between osteoblasts and collagen-bearing ECM. The reciprocal interaction between type I collagen and α2β1 integrin binds the osteoblasts to ECM components. Besides, the promotion of integrins can activate signaling pathways such as the MAPK axis. After the transduction of signals to the nucleus, specific factors such as Runx2 are activated. The concomitant expression of osteoblast marker genes such as OCN-induced osteoblast differentiation (Fig. 6) [128]. It is thought that this model could be a probable mechanism of cell sheet ECM on the host osteoblasts. In another study, it was proposed that the accumulation of extracellular signal-regulated protein kinases-p (P-ERK) occurs in the nucleus after the interaction of integrins with ECM components. Both Runx2 and P-ERK selectively bind to promoters of OCN and bone sialoprotein (BSP). Molecular investigations have revealed that two osteoblastic cis-acting elements, including OSE2a and OSE2b, exist at the proximal promoter of the OCN gene and are essential for OCN expression [129, 130]. It has been indicated that Msx2, Dlx3, and Dlx5 could positively and negatively regulate Runx2 [131]. Runx2 with Dlx3 and Dlx5 could regulate functional osteoblast HD regulatory elements including type I Collagen, OCN, OPN, and BSP [132].

Immunofluorescence imaging indicated that only part of the transplanted BM-MSC sheet directly differentiated into osteocytes and formed the bone matrix [133]. Labeled MSCs in the structure of cell sheets are detectable at the defect sites and in the regenerating tissues about 2 and 8 weeks after transplantation [68]. To show the probable relationship between MSC type and bone formation, Xie et al. used cellular sheets of hES-MSCs and hBM-MSCs. It was found that both MSC sheets, especially hES-MSCs, can differentiate into osteoblasts. The higher regenerative outcomes in animals that received the hES-MSC sheet are possibly due to the secretion of cytokines (Fig. 6) [5].

## Limitations and potential core problems

Besides the benefits and advantages of cell sheets in bone engineering, some related limitations need further consideration [134]. For example, the period required for applied cells to form the sheet structure could be up to four weeks after cell isolation (Table 1). One strategy would be a selection of certain growth factors, appropriate initial cell density, scaffolds, and supportive matrices with specific physicochemical properties that can educate the plated MSCs to produce ECM in the least possible time. Another limiting factor is the short shelf life of cell sheet constructs. It means that at the time that sheets are formed, the patient should be in a suitable condition to receive the graft. The existence of xenobiotic materials such as serum can affect the eligibility of sheet structures in the clinical setting. Using autologous and allogeneic serum for the expansion of MSCs within the cell sheet structure can, in part, but not completely, circumvent the issues associated with the transmission of xenobiotic materials in patients. The variability of the final product is another potential problem that arises from several biological reagents during the fabrication of cell sheets, resulting in varied quality products. Developing standard GMP-grade protocols can reduce several drawbacks related to quality variation. Transporting and the distance between the laboratory and the patient hospitalization should be short. Furthermore, the final products have to be used within two or three days to avoid losing cell-to-cell connection and attachment to the wound beds [134]. For commercialization aspects, the storage, and transportation of products should be carefully addressed. Also, cell sheet manufacturing is a manual process, and the final cell sheet structurally is a very thin, skinny, and sensitive layer. Due to a lack of proper mechanical properties, cells are susceptible to damage [44, 135]. So, the cell sheet process demands an expert operator and educated surgeons to be familiar with the handling of cell sheets [134]. Cryopreservation is another issue in terms of CST. Nowadays, there are few effective methods for cell sheet cryopreservation, thawing, and administration. Regarding these obstacles, cell sheet therapy is restricted to short paths between culture facilities or demands [136].

## Conclusion

Cell sheet engineering is not only limited to in vitro studies but is also used for the regeneration of several soft and hard tissues in animal models and clinical studies. Among various available techniques, the existence of a confluent cell layer, intact ECM, and secretion of several signaling biomolecules make the CST a promising therapeutic approach in CMF bone defects. The combination of CST with other modalities such as nanofibers or other substrates can yield better regenerative outcomes. Despite these descriptions, further investigations are mandatory to improve mechanical properties with a focus on vascularization potential, paracrine activity, and differentiation into the target cell types.

## Data Availability

Not applicable.

## Abbreviations

AD-MSCs: Adipose-derived mesenchymal stem cells
ALP: Alkaline phosphatase
β-TCP: Beta-tricalcium phosphate
BM-MSCs: Bone marrow-derived mesenchymal stem cells
BMP: Bone morphogenetic protein
BRONJ: Bisphosphonate-related osteonecrosis of the jaw
BV/TV: Bone volume to total volume
CMF: Craniomaxillofacial
CSD: Critical size defect
Dex: Dexamethasone
ECM: Extracellular matrix
FGF: Fibroblast growth factor
HA: Hydroxyapatite
hES-MSCs: Human ethmoid sinus mucosa-derived MSCs
hUC-MSCs: Human umbilical cord-derived stem cells
IDO: Immuno-modulator indoleamine 2,3-dioxygenase
IFN: Interferon
IL: Interleukin
IV: Intravenous
MSCs: Mesenchymal stem cells
OCN: Osteocalcin
OMCS: Osteogenic matrix cell sheets
TBI: Tendon–bone interface
TE: Tissue engineering
PLA: Polylactic acid
PLLA: Poly-L-lactide
PLGA: Polylactide-co-glycolide
PRF: Platelet‑rich fibrin granules
UC-MSCs: Umbilical cord-derived stem cells
VEGF: Vascular endothelial growth factor
